# Adjunctive effects of intermittent fasting and exercise with glibenclamide on diabetic nephropathy in rats: a potential role of the polyol pathway

**DOI:** 10.3389/fphys.2025.1683271

**Published:** 2025-11-28

**Authors:** Shereen M. Samir, Hend M. Hassan, Rasha Elmowafy, Neven A. Ebrahim, Emad A. Albadawi, Muayad Albadrani, Wejdan Hussain Owaydhah, Mona G. Elhadidy

**Affiliations:** 1 Department of Medical Physiology, Faculty of Medicine, Mansoura University, Mansoura, Egypt; 2 Department of Human Anatomy and Embryology, Faculty of Medicine, Mansoura University, Mansoura, Egypt; 3 Department of Human Anatomy and Embryology, Faculty of Medicine, New Mansoura University, Mansoura, Egypt; 4 Department of Medical Biochemistry and Molecular Biology, Faculty of Medicine, Mansoura University, Mansoura, Egypt; 5 Department of Medical Biochemistry and Molecular Biology, Faculty of Medicine, Horus University, New Damietta, Egypt; 6 Department of Basic Medical Sciences, College of Medicine, Taibah University, Madinah, Saudi Arabia; 7 Department of Family and Community Medicine and Medical Education, College of Medicine, Taibah University, Madinah, Saudi Arabia; 8 Department of Medical Physiology, Faculty of Medicine, Al-Baha University, Al-Baha, Saudi Arabia

**Keywords:** Bax, Bcl-2, diabetes, gliben, inducible nitric oxide synthase, kidneys, tumor necrosis factor-α, transforming growth factor-β

## Abstract

**Background:**

Diabetic nephropathy (DN) is a major complication of type 2 diabetes, often driven by hyperglycemia-induced activation of the polyol pathway. Exercise and intermittent fasting (IF) are non-pharmacological strategies known to improve glucose homeostasis, yet their renal protective roles remain underexplored.

**Aim:**

To explore how exercise and IF with glibenclamide therapy can have a therapeutic potential in diabetic nephropathy as adjuncts or alternatives to conventional pharmacological treatment by focusing on the polyol pathway as a mechanistic target.

**Methods:**

Type 2 diabetes was induced in rats using an 8-week high-fat diet followed by a single low dose of streptozotocin (STZ). Animals were treated for 4 weeks with glibenclamide (1 mg/kg/day), exercise, IF, or combined triple therapy. Biochemical, molecular, and histopathological analyses were performed to evaluate renal function, oxidative stress, inflammatory mediators, polyol pathway activity, apoptotic markers, and tissue architecture.

**Results:**

Untreated diabetic rats developed hyperglycemia, renal impairment, oxidative stress, and inflammation with marked polyol pathway activation. Triple therapy significantly improved glycemic control, restored antioxidant defenses, reduced pro-inflammatory and apoptotic markers, downregulated transforming growth factor-β (TGF-β) expression, and preserved renal histology.

**Conclusion:**

The combination of glibenclamide, exercise, and IF provides synergistic protection against diabetes-induced nephropathy, primarily through modulation of the polyol pathway, antioxidant enhancement, and suppression of inflammation and fibrosis.

## Highlights

- Diabetic rats showed renal dysfunction, oxidative stress, and polyol pathway activation.
- Triple therapy improved antioxidant defenses and reduced oxidative stress.
- Triple therapy modulated apoptotic and inflammatory markers in renal tissue.
- Renal architecture was preserved by triple therapy in diabetic rats.

## 1 Introduction

An acquired or inherited incapacity of the pancreatic tissue to produce insulin causes diabetes mellitus (DM), a well-known endocrine disorder. According to the report, 300 million individuals are expected to develop diabetes mellitus by 2025 (Nasiry et al., 2017). In diabetes mellitus, the most common pathological outcomes are diabetic nephropathy (DN), neuropathy, and cardiomyopathy. The main consequence of diabetes is diabetes-linked kidney damage, which affects 30%–40% of people with DM even when their diabetes is under control (Nasiry et al., 2019). Diabetic kidney disease is classified using a variety of renal structural dysfunctions, including glomerulosclerosis, mesangial enlargement, thickening of the basement membrane, inflammatory response, and oxidative stress. Therefore, there is an urgent need to identify a more promising drug to treat diabetes mellitus and diabetic nephropathy (Javidanpour et al., 2012; Nasiry et al., 2017).

Glibenclamide (gliben) is the most widely used sulfonylurea drug for the treatment of type 2 diabetes. It causes the plasma membrane of the β cells to depolarize and voltage-gated calcium channels to open in pancreatic β islet cells by blocking ATP-sensitive potassium channels. The β cells release insulin as a result of this calcium influx (Zhang G. et al., 2017).

The practice of eating less food while preserving a minimum level of nutrients is known as food restriction (FR). Among other things, it has been demonstrated to improve pancreatic beta-cell activity and blood glucose regulation (Albosta and Bakke, 2021; Alejandra et al., 2018; Elesawy et al., 2021). Intermittent fasting (IF) is one of the different ways to produce FR (Kunduraci and Ozbek, 2020). Exercise and intermittent fasting have long been recognized as essential non-pharmacological treatments for diabetes because they can improve insulin sensitivity and insulin-stimulated muscle glucose uptake, both of which improve glucose utilization (Harvie and Howell, 2017; Corley et al., 2018; Sutton et al., 2018; Sampath Kumar et al., 2019). Furthermore, they are recognized as adjunctive therapies for the management of type 2 diabetes (Ko et al., 2018).

The two-step metabolic process that turns glucose into fructose is known as the sorbitol or polyol pathway. The pathophysiology of problems in patients with end-stage diabetes is believed to be largely explained by this pathway. The first enzyme in the pathway, aldose reductase (AR), reduces glucose to sorbitol, which is then converted to fructose by sorbitol dehydrogenase (SDH) (Chung et al., 2003). It has been implicated in the enhanced activation of diabetic complications, such as cataracts, retinopathy, neuropathy, and nephropathy. Sorbitol has a physiological osmoprotective function in the kidney as its intracellular level adjusts to the increasing osmolarity of the extracellular fluid along the cortico-papillary axis (Schmolke et al., 1990).

Therefore, the novelty of the study lies in focusing on the polyol pathway as a mechanistic target, comparing exercise and intermittent fasting with glibenclamide therapy in the context of diabetic nephropathy, and exploring the therapeutic potential of lifestyle interventions as adjuncts or alternatives to conventional pharmacological treatment.

## 2 Materials and methods

### 2.1 Chemicals used

Sigma Chemical Co. (St. Louis, MO, United States) provided the streptozotocin (STZ) and gliben. Abcam (Egypt) provided the anti-inducible nitric oxide synthase (iNOS) and anti-tumor necrosis factor-α (TNF-α) antibodies.

### 2.2 Ethical approval

In compliance with NIH and EU animal care standards, the Mansoura University Animal Care and Use Committee (MU-ACUC) provided ethical permission for this experiment (Code: MED.R.23.12.30). The experiment was conducted in the Medical Experimental Research Center (MERC), Faculty of Medicine, Mansoura University, Egypt.

### 2.3 Sample size calculation

Sample size was calculated using G*Power software (version 3.1.9.7). In a one-way ANOVA study, sample sizes of seven rats were obtained from each of the study groups whose means were to be compared.

The total sample of 42 rats will achieve 85% power to detect differences among the means versus the alternative of equal means using an F test with a 0.05 significance level.

### 2.4 Experimental animals

Forty-two adult male Sprague–Dawley rats weighing 180–220 g and 3–4 months old were used in the investigation. Under the observation of a veterinarian, the rats were housed in regulated conditions with a temperature of 22 °C ± 2 °C and a humidity of 55% ± 5%. They were kept in a 12:12-h light and dark cycle following a week of acclimation, during which they were fed diet chow and had unrestricted access to tap water.

### 2.5 Experimental design and induction of type 2 diabetes

**FIGURE 1:**
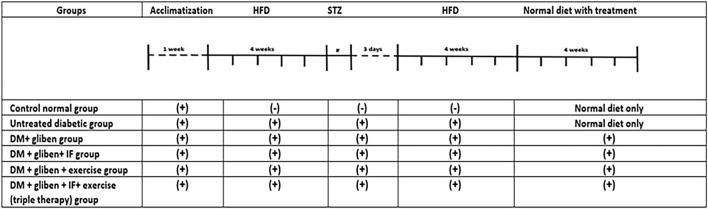
Study design.

Rats were divided into two main groups: a normal control group (7 rats), in which the rats were fed normal chow for 12 weeks, and a high-fat group (35 rats), in which they were fed a high-fat diet composed of 58% fat, 17% carbohydrates, and 25% protein as a proportion of total calories (Al-Megrin et al., 2020). After 4 weeks, the high-fat group received a single dose of STZ (35 mg/kg) dissolved in citrate buffer (pH 4.4) and administered intraperitoneally (i.p.) in the lower right quadrant of the abdomen following a 12-h fast (Srinivasan et al., 2005). To avoid hypoglycemic shock, a 5% glucose solution was administered, followed by a high-fat diet for an additional 4 weeks. Meanwhile, the normal control group received i.p. injections of citrate buffer alone (1 mL/kg) at the same time as STZ administration. Then, blood was drawn from the rats’ tail veins 72 h after STZ administration, and the OneTouch Blood Glucose Monitoring System (LifeScan, Milpitas, United States) was used to measure blood glucose levels. When fasting, blood glucose levels were higher than 300 mg/dL, and high-fat diet (HFD)-STZ developed type II diabetes. The diabetic rats were then arbitrarily split into five groups: untreated diabetic group, DM + gliben [1 mg/kg/day] (Ohadoma and Michael, 2011), DM + gliben + intermittent fasting, DM + gliben + exercise, and DM + gliben + intermittent fasting + exercise (triple therapy). All treatments continued for 4 weeks after the induction of diabetes (Figure 1).

### 2.6 Physiological intervention approaches

#### 2.6.1 Intermittent fasting intervention

The IF started after the induction of DM in the last 4 weeks. The rats in this group underwent a protocol where they were deprived of food for 24 h, followed by an additional 24 h of *ad libitum* access to rat chow. The food was withdrawn or made available at noon every day. There was unlimited access to water throughout the experiment (Hassan HM. et al., 2024).

#### 2.6.2 Exercise intervention

The exercise intervention started after the induction of DM in the last 4 weeks. Every day, swimming was carried out in a large water tank. Exercise was performed by swimming in an iron tank (length 137.5 cm, width 135 cm, and depth 50 cm) containing tap water maintained at 32 °C ± 2 °C. The rats were kept from ever touching the tank’s floor with their feet during the swimming activity. The rats’ swimming program was divided into two stages: training and adaptation. On the final day of the 1 week, swimming time was progressively increased from 10 to 30 min throughout the adaptation phase. The goal of the adaptation stage was to prevent excessive physiological changes and stress caused by water (Minaii et al., 2014). The intense exercise-training phase began after the adaptation period and lasted for 3 weeks, with sessions of 30 min each day, 5 days a week, scheduled at the same time every day (from 9:00 a.m. to 11:00 a.m.) (Marcelino et al., 2013; Stone et al., 2015). The animals were allowed to swim freely, without any extra weight, and were gently stimulated to continue swimming. This protocol has moderate intensity (Seo et al., 2014). Then, rats were kept warm beneath an electric heater and dried after swimming.

### 2.7 Measurement of the body weight of studied rats

The rats’ body weight was measured and documented at the start of the trial. They were also weighed on the 12th week using a digital weighing scale to detect the amount of change in their weight.

### 2.8 Specimen collection

At the end of the 12 weeks, all experimental rats were placed in metabolic cages for 24 h following treatment and the conclusion of the experiment, during which urine was collected for additional measurements. Sodium thiopental (35 mg/kg, intraperitoneally) was then used to anesthetize the rats (Zhou X. et al., 2020). Additionally, blood samples were collected directly from the tail vein and stored at −20 °C until analysis. Both kidneys were also meticulously dissected, and the right kidney was preserved in 10% formalin solution for histopathological and immunohistochemical examinations. The left kidney was divided into two parts. One part was homogenized in phosphate-buffered saline solution (0.1 M PBS, pH 7.4). After centrifuging the homogenates for 30 min at 4 °C at 10,000 rpm, the supernatants were maintained at −80 °C until the biochemical tests (Tawfeek et al., 2021). As soon as feasible, the other portion was fully immersed in RNA Later Stabilization Solution (10 μL per 1 mg tissue sample) (QIAGEN, Germany) at 4 °C overnight before being stored at −80 °C until the isolation of total RNA and subsequent real-time reverse transcription polymerase chain reaction (RT-PCR).

### 2.9 Biochemical studies

#### 2.9.1 Measurement of fasting blood glucose and insulin levels and homeostatic model assessment of insulin resistance (HOMA-IR)

The glucose oxidase method (CONTOUR NEXT, United States) was used to measure the rats’ blood glucose levels after they had fasted for 24 h before the experiment’s conclusion. An insulin ELISA kit (Diagnostic Automation, United States) was used to measure insulin levels by the sandwich ELISA method during fasting. HOMA-IR was calculated using the following formula: (fasting blood glucose levels × fasting blood insulin levels)/405 (Muniyappa et al., 2009).

#### 2.9.2 Measurement of lipid profile in serum samples of the studied groups

The concentrations of triglycerides (TGs), total cholesterol, HDL-cholesterol (HDL-C), and LDL-cholesterol (LDL-C) were assessed in the serum using colorimetric commercial kits purchased from Spinreact, Spain, after following the manufacturer’s instructions; absorbance was measured spectrophotometrically at 505 nm. LDL-C (mg/dL) = Total cholesterol–HDL-C – (Triglycerides/5) (Fossati and Prencipe, 1982; Wieland and Seidel, 1983).

#### 2.9.3 Assessment of renal parameters in blood and urine samples of the studied groups

Serum samples were obtained, and after following the manufacturer’s instructions, the commercial colorimetric kits acquired from Biodiagnostic (Cairo, Egypt) were used to assess kidney function parameters such as creatinine, albumin, and blood urea nitrogen (BUN). The urine samples were centrifuged at 3,000 rpm for 5 min at 4 °C. After that, the supernatant was collected and stored −20 °C until analysis. The ERBA CHEM-7 equipment (ERBA Diagnostics, India) and commercially available kits (Biodiagnostic, Egypt) were used to measure urine parameters such as microalbumin, total protein, and creatinine (Guo J. et al., 2021).

Creatinine clearance (Cr Cl) in rats can be calculated using the following formula:

$$\text{Cr Cl }\left(\text{mL}/\min\right)=\left(U\_\text{cr}\times V\right)\;/\;\left(P\_\text{cr}\times t\right),$$

where

- U_cr represents the urine creatinine concentration (mg/dL or µmol/L);
- V represents the urine volume (mL);
- P_cr represents the plasma creatinine concentration (mg/dL or µmol/L); and
- t represents the urine collection time (minutes) (Zhang S. et al., 2017).

#### 2.9.4 Measurement of oxidative stress markers in the renal tissue homogenate

Using commercial colorimetric kits acquired from a biodiagnostic firm in Cairo, Egypt, the levels of reduced glutathione (GSH), superoxide dismutase (SOD), malondialdehyde (MDA), and catalase (CAT) in the renal tissue homogenates were measured. Following the manufacturer’s instructions, absorbance was measured spectrophotometrically at various wavelengths using the colorimetric method. Furthermore, the oxidative DNA damage parameter, 8-hydroxy-2ʹ-deoxy-guanosine (8-OHdG) was measured using a kit from MyBioSource, United States (Cat. No. MBS267513). Following the manufacturer’s recommendations, this kit employed the “double antibody sandwich” technique, where the OD was read at 450 nm using a plate reader (Kumar et al., 2014).

#### 2.9.5 Measurement of inflammatory mediators in the renal tissue homogenate

Pro-inflammatory cytokines such as nuclear factor kappa B (NF-κB), interleukin-1β (IL-1β), interleukin-6 (IL-6), and TNF-α, and the anti-inflammatory interleukin-10 (IL-10) were measured in renal tissue homogenate using the sandwich ELISA method with kits from MyBioSource, United States (Cat. No. MBS175904, MBS2023030, MBS2021530, MBS453975, and MBS2707969, respectively). Following the manufacturer’s instructions, the color change was measured spectrophotometrically at a wavelength of 450 nm using a plate reader (Guo J. et al., 2021).

#### 2.9.6 Measurement of aldose reductase and sorbitol dehydrogenase activities and sorbitol levels in the renal tissue homogenate

The activity of the aldose reductase enzyme was determined in renal tissue homogenate by colorimetric assay using a kit from Abcam, United States (Cat. No. ab273276), which measures the ability of aldose reductase to catalyze the oxidation of NADPH. The reaction progress was followed by monitoring the decrease in absorbance at 340 nm using a microplate reader.

Sorbitol dehydrogenase enzyme activity was determined in the renal tissue homogenate by colorimetric assessment methods using a kit from Abcam, United States (Cat. No. ab252902). In this assay, sorbitol dehydrogenase utilized a provided substrate while reducing NAD + to form NADH. NADH reacted with the developer, leading to the formation of a chromophore with strong absorbance at OD 450 nm.

Furthermore, sorbitol level was assessed by colorimetric method using the D-Sorbitol Assay Colorimetric Kit (Cat. No. BA0147) from Assay Genie, Ireland, which involved an end-point enzyme-coupled MTT/NAD reaction that formed a colored product with an absorption maximum at 565 nm. The increase in absorbance at 565 nm is directly proportional to the sorbitol concentration.

### 2.10 mRNA quantification by quantitative real-time reverse transcription-PCR

Renal tissue samples were homogenized using liquid nitrogen. Following the manufacturer’s recommendations, total RNA was then extracted from renal tissue using the QIAzol reagent (QIAGEN, Germany). The quantities and purities of RNA yield were measured using Thermo Fisher Scientific NanoDrop One (United States). One microgram of RNA was converted into first-strand cDNA using a ProFlex Thermal Cycler (Applied Biosystems, United States) and the RevertAid First-Strand cDNA Synthesis Kit (Thermo Fisher scientific, United States).

The cDNA templates were amplified using a real-time PCR device (Azure Cielo 6, United States). The 20 μL total reaction volume for the amplification procedure included 10 μL of HERA SYBR Green PCR Master Mix (Bioline, United Kingdom), 1 μL of cDNA template, 2 μL (10 pmol/μL) gene primer, and 7 μL of nuclease-free water. The thermal profile started with an initial denaturation at 95 °C for 2 min, followed by 40 cycles of annealing and extension at 60 °C for 30 s.

The glyceraldehyde-3-phosphate dehydrogenase (*GAPDH*) gene was used as a reference gene to normalize RNA expression levels. The primer pair sequences utilized in this study are shown in Table 1. The primer sets were designated using Primer3Plus software (https://www.bioinformatics.nl/cgi-bin/primer3plus/primer3plus.cgi). Additionally, the Primer-BLAST program (https://www.ncbi.nlm.nih.gov/tools/primer-blast/) was used to assess primers’ specificity. The primer sets were provided by Vivantis, a Malaysian company. Melting curve analysis was used to confirm the specificity of the PCR results. Relative gene expression levels were expressed using the formula ΔCt = Ct target gene − Ct housekeeping gene. The fold change in gene expression was then calculated using the 2^−ΔΔCT^ technique (Livak and Schmittgen, 2001).

**TABLE 1:** Rat primer sequences used in qRT-PCR investigation.

Gene	Sequence	Product size	RefSeq	Reference
*BAX*	F: AAGAAGCTGAGCGAGTGTCT	361 bp	NM_017059.2	Albadawi et al. (2024)
	R: CAAAGATGGTCACTGTCTGC			
*BCL-2*	F: GTACCTGAACCGGCATCT	97 bp	NM_016993.1	Elsayed et al. (2021)
	R: ATCAAACAGAGGTCGCA			
*TGF-β*	F: TGCTAATGGTGGACCGCAA	101 bp	NM_021578.2	Bio Informatics (2024)
	R: CACTGCTTCCCGAATGTCTGA			
*Glyceraldehyde-3-phosphate dehydrogenase (GAPDH)*	F: TGGGAAGCTGGTCATCAAC	78 bp	NM_017008.4	Albadawi et al. (2024)
	R: GCATCACCCCATTTGATGTT			

### 2.11 Histopathological and immunohistochemical staining

Hematoxylin and eosin (H&E) staining was performed on renal specimens after they were sliced, preserved, and embedded in paraffin. In the kidney, immunodetection was performed for TNF-α and iNOS markers. Paraformaldehyde (4%) was used to fix the tissues, 0.2% Triton X-100 was used to stabilize them, and nonspecific receptors were blocked for 30 min using normal goat serum mixed in phosphate-buffered saline (PBS). They were then exposed to the primary antibodies, iNOS rabbit monoclonal (ab283655; 1:100 dilution, Abcam) and TNF-α rabbit monoclonal (ab39162; 1:100 dilution, Abcam), for 12 h at 4 °C. Following a wash with 0.1% PBS/bovine serum albumin (BSA), the samples were incubated for 2 h at room temperature with the corresponding secondary antibodies at the recommended dilution (1:50). Browning was achieved using streptavidin–peroxidase and diaminobenzidine (DAB). After 30 s of Mayer’s hematoxylin counterstaining, three PBS washes and one distilled water wash were performed. After mounting the slides, an Olympus microscope (CX41) was used to examine them (Hassan H. M. et al., 2024).

### 2.12 Histopathological scoring

In the present study, we evaluated the histopathological changes, represented by glomerular thickening, tubular dilatation, mesangial hypercellularity, and interstitial inflammation, in all studied groups. A semi-quantitative score was assigned based on the absence (=0) or presence (=1) of each of the histopathological finding, and a total score was calculated for each section.

### 2.13 Morphometric studies

Each rat’s five randomized, non-overlapping fields were analyzed. The NIH (National Institute of Health) ImageJ tool was used to calculate the area percentage of the brown immune-stained TNF-α and iNOS sections for each group (×400). ImageJ software was used to separate the brown pixels that had been immunohistochemically stained from the background. The color threshold scale for the immunohistochemically stained brown patches was established by adjusting the brightness and saturation of the chosen images. Finally, the selected photos’ positively stained color pixels (DAB) were separated from the background (H&E-stained pixels) using the preset threshold (Ebrahim et al., 2024).

### 2.14 Statistical analysis

For the statistical study, GraphPad Prism 10 (GraphPad Software Inc., CA, United States) was used. Data were tested for normality using the Shapiro–Wilk test. Comparisons involving more than two groups were analyzed using a one-way ANOVA followed by pairwise comparisons using the Dunnett’s multiple comparisons test. The numerical data were displayed as the mean ± standard deviation, and *p* ≤ 0.05 was considered statistically significant.

## 3 Results

### 3.1 Impact of exercise and intermittent fasting on the study groups’ body weight

The HFD group’s body weight significantly decreased compared to the control and treatment groups, as shown in Table 2. Furthermore, compared to the other treatment groups, the HFD co-treated with exercise, IF, and gliben showed a significant decrease in body weight (*p* < 0.0001, *p* < 0.001, and *p* < 0.01, respectively).

**TABLE 2:** Body weights in different treatment groups.

Body weight	Negative control	HFD	HFD + gliben	HFD + gliben + exercise	HFD + gliben + IF	HFD + gliben + exercise + IF
W0 Body weight (g)	152.5 ± 3.5	157.5 ± 2.1	152.5 ± 0.7	159 ± 1.4	157 ± 7.1	154 ± 4.2
W12 Body weight (g)	274.5 ± 3.3	206.7 ± 5.7 ****	282.8 ± 3.1 *** ####	252.8 ± 2.5 **** #### $$	244 ± 2.9 **** ### $$$	274 ± 2.4 ****

Data were analyzed using an ANOVA test (Prism 10 software) to compare HFD versus treated groups at W0 and W12 and HFD + Gliben + IF + exercise group and other treatment groups at the same time intervals. Significant differences were observed between the control and treatment groups compared to the HFD group (****, *p* < 0.0001; ***, *p* < 0.001), between the HFD group co-treated with exercise + IF and gliben compared to the other treated groups (####, *p* < 0.0001; ###, *p* < 0.001), and between gliben + exercise or gliben + IF compared to gliben-alone treatment group ($$$, *p* < 0.001; $$, *p* < 0.01). Data are expressed as the mean ± standard deviation, and *p* ≤ 0.05 was considered statistically significant.

HFD, high-fat diet; IF, intermittent fasting; W0, 0 week; W12, 12 weeks.

The gliben + exercise or gliben + IF groups experienced a substantial increase in body weight in week 12 of the experiment compared to the gliben-alone treatment group (*p* < 0.01 and *p* < 0.05, respectively).

### 3.2 HOMA-IR and the effects of exercise and intermittent fasting on insulin and fasting blood glucose levels

**FIGURE 2:**
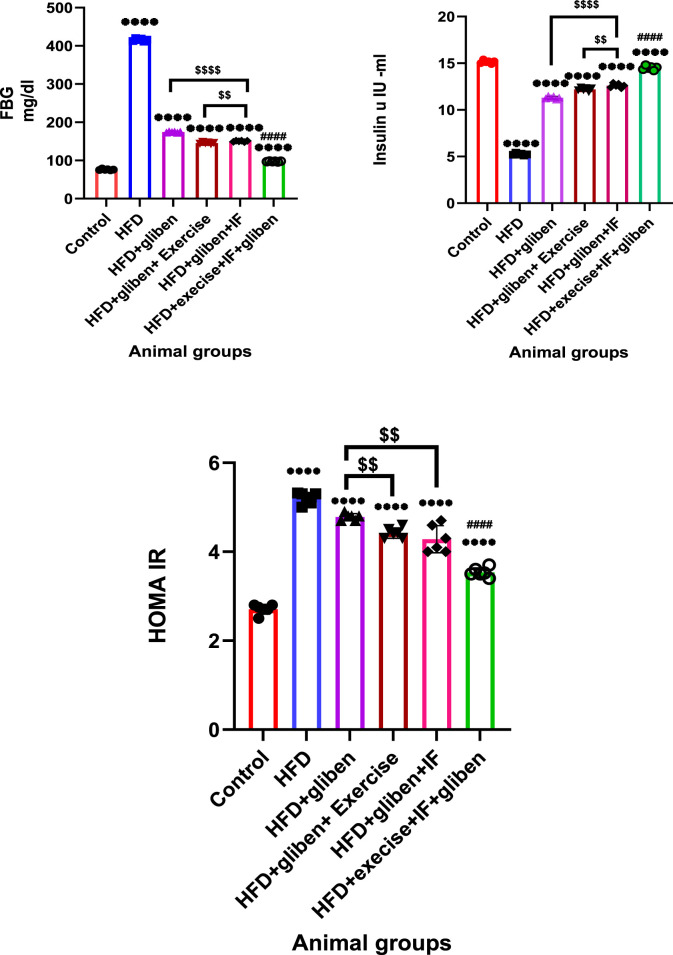
Effect of intermittent fasting and exercise on FBG and insulin levels and HOMA-IR: GraphPad Prism 10 was used to perform the statistical analysis (GraphPad Software Inc., CA, United States). For comparisons between more than two groups, a one-way ANOVA and Dunnett’s multiple comparisons test were used, and for comparisons between each pair of groups, an unpaired t-test was used. *p* < 0.05 was used to indicate statistical significance, and the data were presented as the mean ± standard deviation. The data showed significant differences between the control and treatment groups compared to the HFD group (****, *p* < 0.0001), and between the HFD co-treated with exercise + IF and gliben compared to other treatment groups (####, *p* < 0.0001), and between the gliben + exercise or gliben + IF compared to the gliben-alone treatment group ($$, *p* < 0.01; $, *p* < 0.05).

As illustrated in Figure 2, the HFD group’s fasting insulin levels significantly decreased, while their fasting blood glucose (FBG) and HOMA-IR levels significantly increased compared to the control and treatment groups (*p* < 0.0001). Compared to the other treatment groups, these outcomes were considerably (*p* < 0.0001) reversed in the triple therapy group. Additionally, when gliben was administered with either exercise or IF, the gliben-alone treatment group showed significantly lower FBG and HOMA-IR levels (*p* < 0.01) and higher fasting insulin levels (*p* < 0.05).

### 3.3 Impact of exercise and intermittent fasting on lipid profile in the blood samples of the groups under study

**FIGURE 3:**
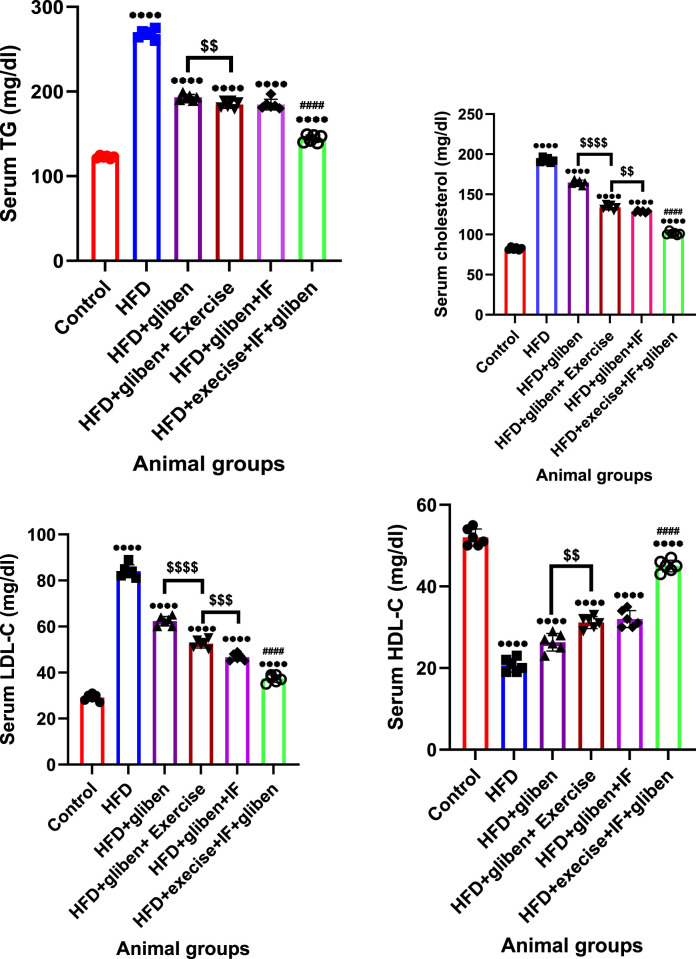
Effect of intermittent fasting and exercise on lipid profile in blood samples of studied groups: GraphPad Prism 10 was used to perform the statistical analysis (GraphPad Software Inc., CA, United States). For comparisons between more than two groups, a one-way ANOVA and Dunnett’s multiple comparisons test were used, and for comparisons between each pair of groups, an unpaired t-test was used. *p*< 0.05 was used to indicate statistical significance, and the data were presented as the mean ± standard deviation. The data showed statistically significant differences between the control and treatment groups compared to the HFD group (****, *p*< 0.0001), between HFD co-treated with exercise + IF and gliben compared to other treatment groups (####, *p*< 0.0001, ##, *p*< 0.01, and #, *p*< 0.05), and between gliben + exercise or gliben + IF compared to the gliben-alone treatment group ($$, *p*< 0.01; $, *p*< 0.05).

Figure 3 demonstrated that, compared to the control and treatment groups, the HFD group’s levels of serum TG, cholesterol, and LDL were significantly increased, while HDL was significantly reduced (*p* < 0.0001). In contrast to other treatment groups, the triple therapy group exhibited a significant decrease in serum TG, cholesterol, and LDL levels and a significant increase in HDL levels (*p* < 0.0001). Furthermore, gliben + exercise or gliben + IF significantly reduced serum TG, cholesterol, and LDL levels while significantly increasing HDL levels compared to the gliben-alone treatment group (*p* < 0.01).

### 3.4 Impact of exercise and intermittent fasting on renal parameters in the blood and urine samples of the groups under study

**FIGURE 4:**
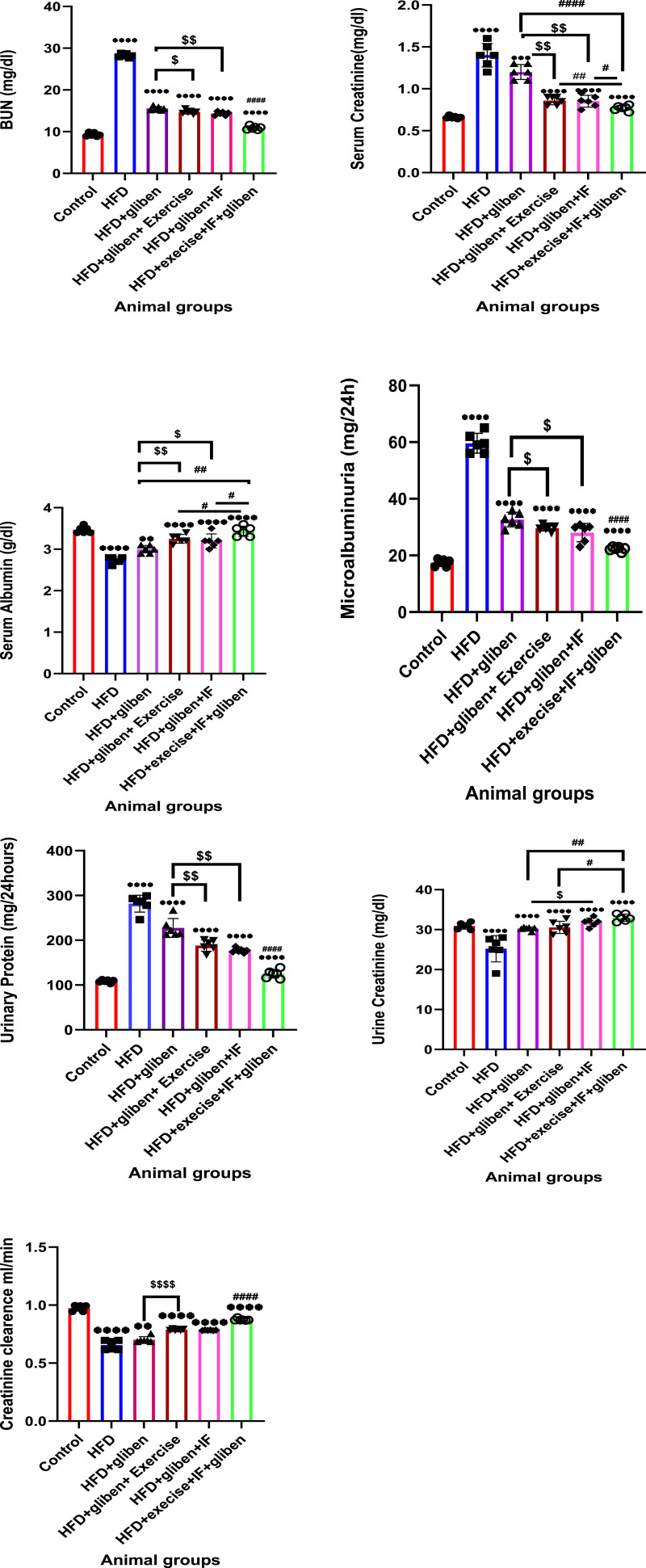
Effect of intermittent fasting and exercise on renal parameters in blood and urine samples of studied groups: GraphPad Prism 10 was used to perform the statistical analysis (GraphPad Software Inc., CA, United States). For comparisons between more than two groups, a one-way ANOVA and Dunnett’s multiple comparisons test were used, and for comparisons between each pair of groups, an unpaired t-test was used. *p*< 0.05 was used to indicate statistical significance, and the data were presented as the mean ± standard deviation. The data showed statistically significant differences between the control and treatment groups compared to the HFD group (****, *p*< 0.0001), between HFD co-treated with exercise + IF and gliben compared to other treatment groups (####, *p*< 0.0001, ##, *p*< 0.01, and #, *p*< 0.05), and between gliben + exercise or gliben + IF compared to the gliben-alone treatment group ($$, *p*< 0.01; $, *p*< 0.05).(a).

As shown in Figure 4, the HFD group’s BUN and serum creatinine levels were considerably greater than those of the control and treatment groups (*p* < 0.0001). The combined HFD + gliben + IF + exercise group had significantly lower BUN and serum creatinine levels than the other treatment groups (*p* < 0.0001). Furthermore, compared to the gliben-alone therapy group, gliben + exercise or gliben + IF significantly decreased serum creatinine and BUN levels (*p* < 0.01; *p* < 0.05).

The serum albumin level was significantly lower (*p* < 0.0001) in the HFD group than that in the control and treatment groups. The triple therapy group’s serum albumin levels were significantly greater than those of the other treatment groups (*p* < 0.01). Additionally, compared to the gliben-only therapy group, gliben plus either exercise or IF significantly increased serum albumin levels (*p* < 0.01; *p* < 0.05).

Additionally, when the HFD group was compared to the control and treatment groups, the data showed a significant increase in urinary microalbumin and total protein levels (*p* < 0.0001) and a significant decrease in urine creatinine levels (*p* < 0.0001). These results were much better in the triple therapy group than in the other treatment groups (*p* < 0.0001). Compared to the HFD + gliben + exercise group, the triple treatment group’s urinary creatinine levels increased less significantly (*p* < 0.05). Additionally, the combination of gliben and either exercise or IF led to a significant decrease in urinary microalbumin and total protein levels (*p* < 0.01 and *p* < 0.05, respectively) and a significant increase in urinary creatinine levels (*p* < 0.01 and *p* < 0.05) compared to the group treated with gliben alone.

Moreover, the creatinine clearance demonstrated a significant difference across the study groups. It was significantly improved in the treatment groups compared to the untreated HFD group, with marked superiority for the triple treatment group over the other treatment groups.

### 3.5 Impact of exercise and intermittent fasting on oxidative stress indicators in the research groups’ kidney tissue

**FIGURE 5:**
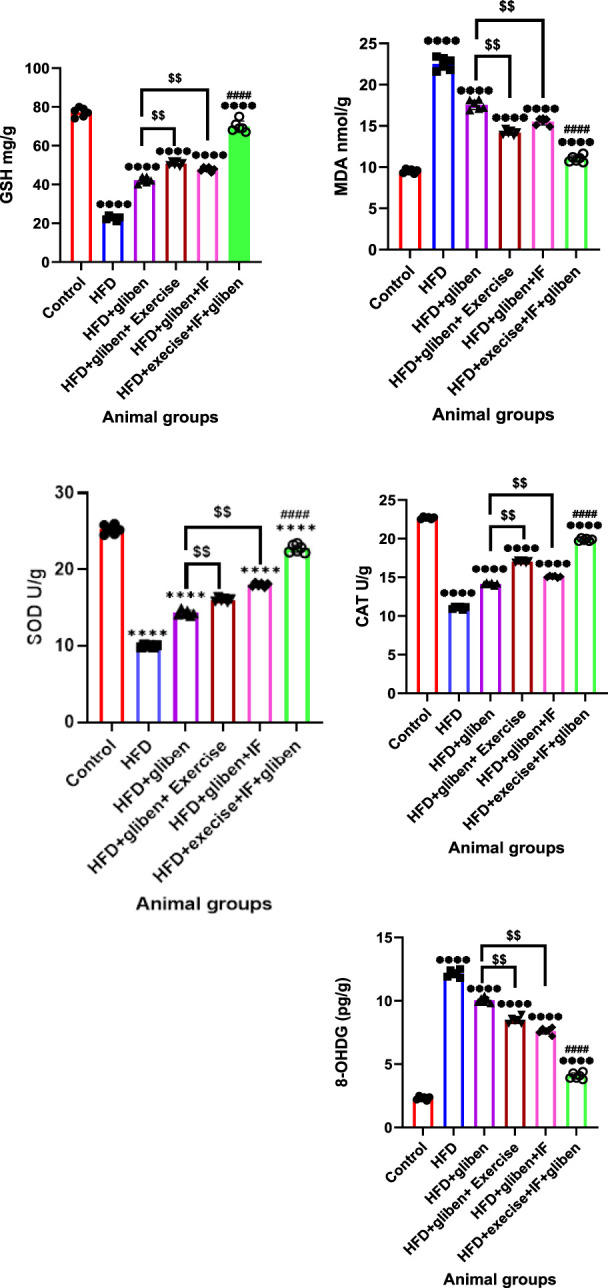
Effect of intermittent fasting and exercise on oxidative stress markers in renal tissue of the study groups: GraphPad Prism 10 was used to perform the statistical analysis (GraphPad Software Inc., CA, United States). For comparisons between more than two groups, a one-way ANOVA and Dunnett’s multiple comparisons test were used, and for comparisons between each pair of groups, an unpaired t-test was used. *p* < 0.05 was used to indicate statistical significance, and the data were presented as the mean ± standard deviation. The data showed significant differences between the control and treatment groups compared to the HFD group (****, *p* < 0.0001), between the HFD co-treated with exercise + IF and gliben compared to other treatment groups (####, *p* < 0.0001), and between the gliben + exercise or gliben + IF compared to the gliben-alone treatment group ($$, *p* < 0.01; $, *p* < 0.05).

Figure 5 shows that, compared to the control and treatment groups, the HFD group’s levels of MDA and 8-OHdG significantly increased, while GSH, CAT, and SOD levels significantly reduced (*p* < 0.0001). In contrast to other treatment groups, the triple therapy group exhibited a significant decrease in MDA and 8-OHdG levels and a significant increase in GSH, CAT, and SOD levels (*p* < 0.0001). Furthermore, gliben + exercise or gliben + IF significantly reduced MDA and 8-OHdG levels while significantly increasing GSH, CAT, and SOD levels compared to the gliben-alone treatment group (*p* < 0.01).

### 3.6 Impact of exercise and intermittent fasting on the levels of inflammatory mediators in the research groups’ kidney tissue

**FIGURE 6:**
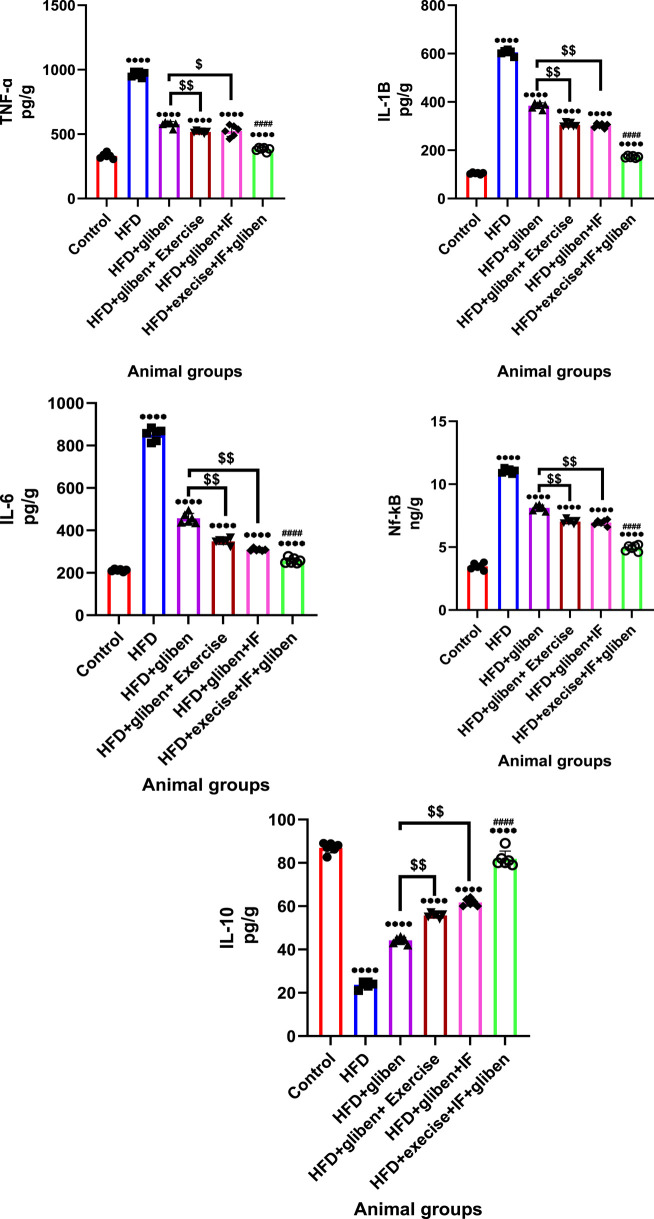
Effect of intermittent fasting and exercise on inflammatory mediators in renal tissue of the study groups: GraphPad Prism 10 was used to perform the statistical analysis (GraphPad Software Inc., CA, United States). A one-way ANOVA and Dunnett’s multiple comparisons test were used for comparisons between more than two groups, and for comparisons between each pair of groups, an unpaired t-test was used. *p*< 0.05 was used to indicate statistical significance, and the data were presented as the mean ± standard deviation. The data showed significant differences between the control and treatment groups compared to the HFD group (****, *p*< 0.0001), between the HFD co-treated with exercise + IF and gliben compared to other treatment groups (####, *p*< 0.0001), and between the gliben + exercise or gliben + IF compared to the gliben-alone treatment group ($$, *p*< 0.01; $, *p*< 0.05).(a).

Comparing the HFD group to the control and treatment groups (Figure 6) demonstrated a significant decrease in the anti-inflammatory marker IL-10 level and a significant increase in TNF-α, IL-1, IL-6, and NF-κB levels (*p* < 0.0001). In addition, compared to other treatment groups, the triple therapy group exhibited a significant increase in IL-10 levels and a significant decrease in TNF-α, IL-1κ, IL-6, and NF-κB levels (*p* < 0.0001). Furthermore, those levels were significantly different in the groups treated with gliben plus either exercise or IF than in the group treated with gliben alone (*p* < 0.01).

### 3.7 Effects of intermittent fasting and exercise on aldose reductase and sorbitol dehydrogenase activities and sorbitol level in renal tissue from the studied groups

**FIGURE 7:**
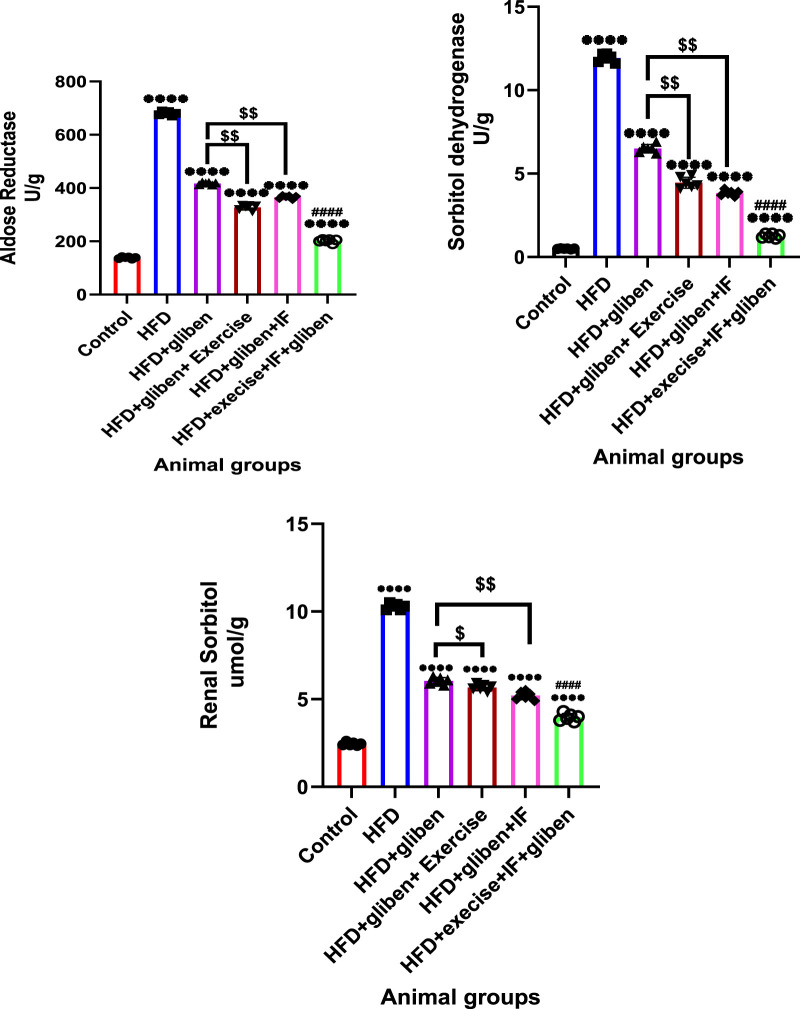
Effect of intermittent fasting and exercise on aldose reductase and sorbitol dehydrogenase activity and renal sorbitol level in renal tissue of the study groups: GraphPad Prism 10 was used to perform the statistical analysis (GraphPad Software Inc., CA, United States). A one-way ANOVA and Dunnett’s multiple comparisons test were used for comparisons between more than two groups, and for comparisons between each pair of groups, an unpaired t-test was used. *p* < 0.05 was used to indicate statistical significance, and the data were presented as the mean ± standard deviation. The data showed significant differences between the control and treatment groups compared to the HFD group (****, *p* < 0.0001), between the HFD co-treated with exercise + IF and gliben compared to other treatment groups (####, *p* < 0.0001), and between the gliben + exercise or gliben + IF compared to the gliben-alone treatment group ($$, *p* < 0.01; $, *p* < 0.05).

Figure 7 shows that the HFD group had considerably greater sorbitol and enzyme activity levels of aldose reductase and sorbitol dehydrogenase than the control and treatment groups (*p* < 0.0001). Furthermore, sorbitol levels and enzyme activity were significantly lower in the triple therapy group than in the other treatment groups (*p* < 0.0001). Compared to the group treated with gliben alone, the group treated with gliben plus either exercise or IF had significantly lower levels of sorbitol and enzyme activity (*p* < 0.01; *p* < 0.05).

### 3.8 Effects of intermittent fasting and exercise on BAX, BCL-2, and TGF-β gene expression in renal tissue from the studied groups

**FIGURE 8:**
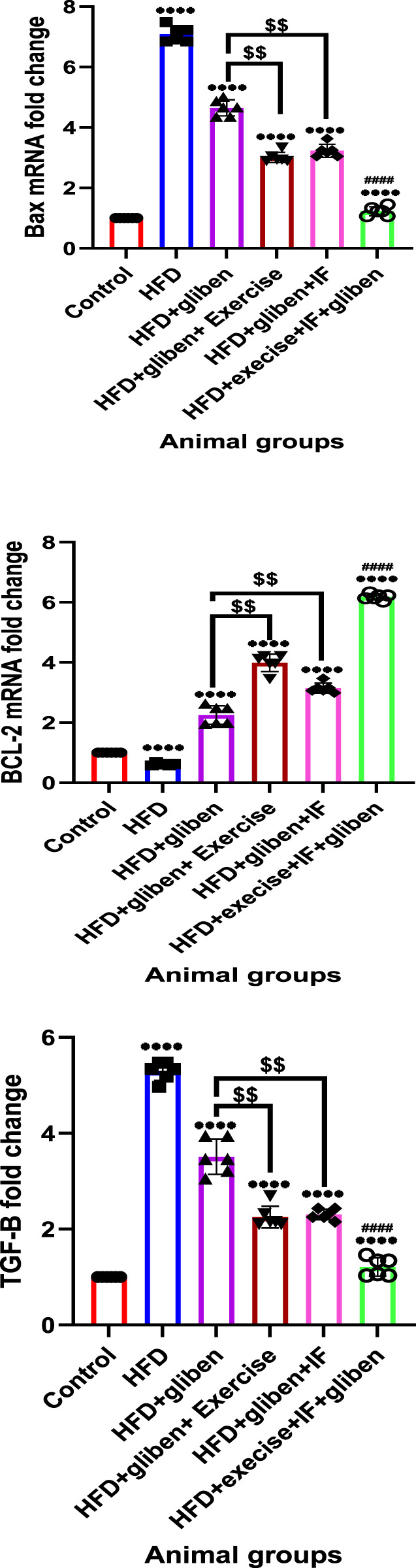
Effects of intermittent fasting and exercise on BAX, BCL-2, and TGF-β gene expression in renal tissue from the studied groups: The unpaired t-test and one-way ANOVA followed by Dunnett’s multiple comparisons test were used. Data are presented as the mean ± standard deviation. Statistical significance was defined as a *p* value < 0.05.

Compared to the control and treatment groups, the HFD group showed a substantial decrease in BCL-2 gene expression (*p* < 0.0001) and a significant increase in BAX and TGF-β (Figure 8). In contrast to other treatment groups, the triple therapy group exhibited a significant increase in BCL-2 gene expression and a significant decrease in BAX and TGF-β (*p* < 0.0001). Additionally, gliben plus either exercise or IF led to a significant increase in BCL-2 gene expression and a significant decrease in BAX and TGF-κ gene expression compared to the gliben-alone treatment group (*p* < 0.01).

### 3.9 Histopathological assessment of renal tissue sections in the study groups

**FIGURE 9:**
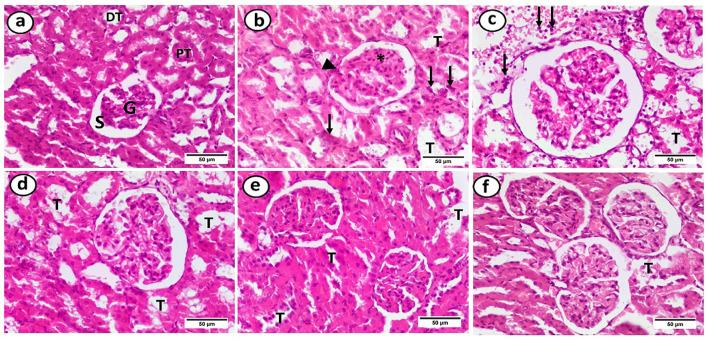
Photomicrographs of paraffin sections in the renal cortex. **(a)** The control group showed that Bowman’s space (S) surrounds the glomerulus (G), proximal tubules (PTs), and distal tubules (DTs). **(b)** The HFD group displayed glomerular alterations, including segmental thickening of the glomerular basement membrane (arrowhead), hypercellularity and growth of the mesangial matrix (*), tubular dilatation (T), and diffuse inflammatory infiltration in the renal interstitium (arrows). **(c)** The HFD + gliben group displayed a dispersed inflammatory cellular infiltration in the interstitium (arrows) and dilatation of most of the renal tubules (T). **(d)** The majority of tubules (T) in the HFD + gliben + exercise group displayed dilatation. **(e)** Some tubules (T) in the HFD + gliben + IF group were dilated. **(f)** Aside from some tubular dilatation (T), the HFD + gliben + IF + exercise group displayed normal architecture. **(d–f)** These groups lacked the glomerular alterations and the inflammatory infiltrate (H&E, ×400).

Sections of the HFD group’s renal cortical tissue revealed a diffuse inflammatory infiltration in the renal interstitium, tubular dilatation, mesangial matrix enlargement, hypercellularity, and segmental thickening of the glomerular basement membrane. When gliben was administered, some tubules also displayed a deformed shape. Except for some tubular deformation, the HFD + gliben + IF + exercise group showed normal architecture (Figure 9).

### 3.10 Effects of intermittent fasting and exercise on the renal cortical tissue expression of iNOS protein in the study groups

**FIGURE 10:**
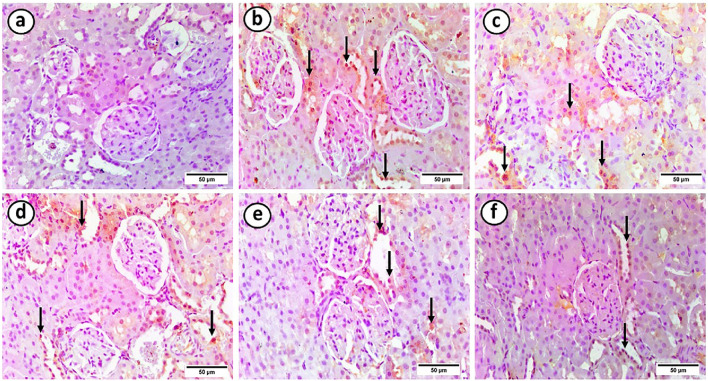
Photomicrographs of paraffin sections in the renal cortex. **(a)** The control group showed a negative reaction. **(b)** The majority of renal tubules (arrows) displayed a robust positive reaction in the HFD group. **(c)** The majority of renal tubules (arrows) in the HFD + gliben group exhibited a moderately positive reaction. **(d)** The majority of renal tubules (arrows) in the HFD + gliben + exercise group exhibited a moderately positive reaction. **(e)** The majority of the renal tubules (arrows) in the HFD + gliben + IF group exhibited a moderately positive reaction. **(f)** Most renal tubules (arrows) in the HFD + gliben + IF + exercise group exhibited a mildly favorable reaction (iNOS, ×400).

The control group showed negative iNOS immunostaining. However, the HFD group showed strong positive immunostaining in most renal tubules. On the other hand, the HFD + gliben, HFD + gliben + exercise, and HFD + gliben + IF groups showed moderate positive immunostaining in most renal tubules. However, the triple therapy group showed mild positive immunostaining in most renal tubules (Figure 10).

### 3.11 Effects of intermittent fasting and exercise on renal cortical tissue expression of TNF-α in the study groups

**FIGURE 11:**
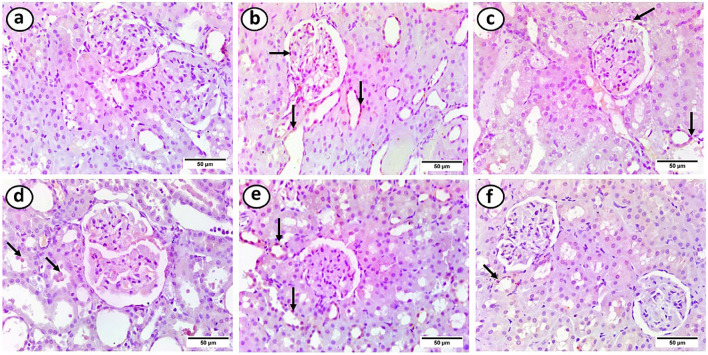
Photomicrographs of paraffin sections in the renal cortex. **(a)** The control group showed a negative reaction. **(b)** The inner layer of the glomerulus and most renal tubules displayed positive immunostaining in the HFD group (arrows). **(c)** The inner layer of the glomerulus and the majority of renal tubules had a positive response in the HFD + gliben group (arrows). **(d)** The majority of renal tubules (arrows) responded favorably to the HFD + gliben + exercise group. **(e)** The majority of renal tubules (arrows) in the HFD + gliben + IF group exhibited a positive reaction. **(f)** Some renal tubules (arrows) in the HFD + gliben + IF + exercise group exhibited a favorable reaction (TNF-α, ×400).

The control group showed negative TNF-α immunostaining. However, the HFD group showed a positive reaction in the majority of renal tubules and the inner layer of the glomerulus. On the other hand, the HFD + gliben group showed positive immunostaining in most renal tubules and the inner layer of the glomerulus. The HFD + gliben + exercise and HFD + gliben + IF groups showed positive immunostaining in most renal tubules. However, the triple therapy group showed a positive reaction in some renal tubules (Figure 11).

### 3.12 Histopathological scoring results

**FIGURE 12:**
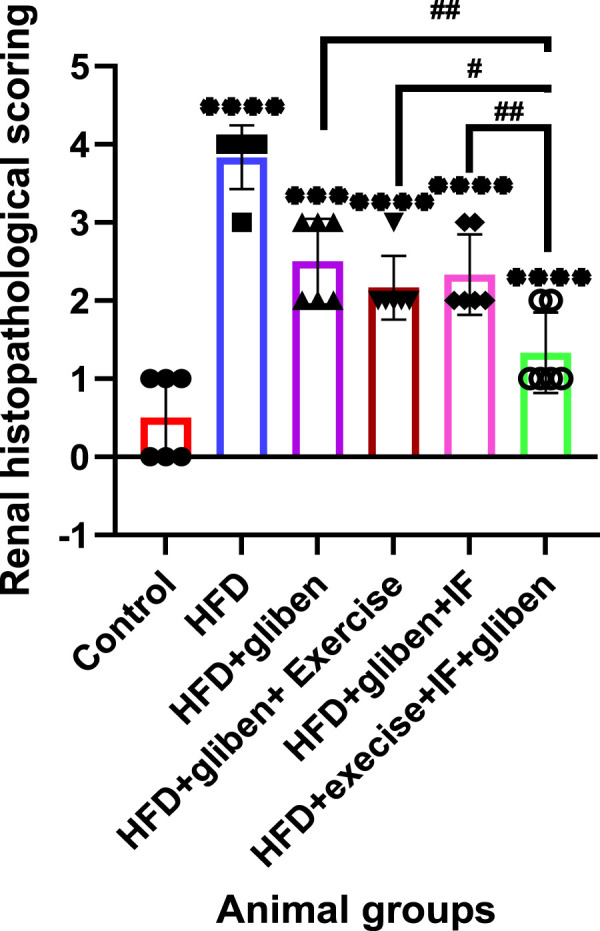
Renal histopathological scoring: GraphPad Prism 10 was used to perform the statistical analysis (GraphPad Software Inc., CA, United States). For comparisons between more than two groups, a one-way ANOVA and Dunnett’s multiple comparisons test were used, and for comparisons between each pair of groups, an unpaired t-test was used. *p* < 0.05 was used to indicate statistical significance, and the data were presented as the mean ± standard deviation. The results showed that the control and treatment groups differed statistically significantly from the HFD group (****, *p* < 0.0001; **, *p* < 0.01), the HFD co-treated with exercise + IF and gliben differed significantly from other treatment groups (####, *p* < 0.0001; ###, *p* < 0.001; and #, *p* < 0.05), and the gliben + exercise or gliben + IF differed significantly from the gliben-alone treatment group ($$, *p* < 0.01; $, *p* < 0.05). The gliben + exercise and gliben + IF groups did not differ significantly.

Figure 12 shows a descriptive analysis of various histopathological changes, illustrating the negative effects of an HFD and the positive effects of gliben, exercise, and IF. The scores for the histopathological changes (sum of scores in the six examined fields) were significantly higher in the HFD group than in the control and treatment groups. Interestingly, the HFD co-treated with exercise + IF and gliben differed significantly from other treatment groups.

### 3.13 Effects of intermittent fasting and exercise on renal tissue iNOS and TNF-α area percentage of positive reaction in the study groups

**FIGURE 13:**
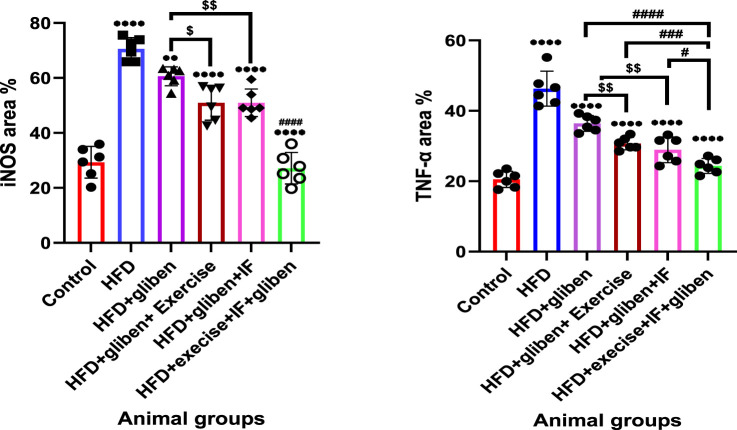
Area % of positive iNOS and TNF-α reaction: GraphPad Prism 10 was used to perform the statistical analysis (GraphPad Software Inc., CA, United States). For comparisons between more than two groups, a one-way ANOVA and Dunnett’s multiple comparisons test were used, and for comparisons between each pair of groups, an unpaired t-test was used. *p* < 0.05 was used to indicate statistical significance, and the data were presented as the mean ± standard deviation. The results showed that the control and treatment groups differed statistically significantly from the HFD group (****, *p* < 0.0001; **, *p* < 0.01), the HFD co-treated with exercise + IF and gliben differed significantly from other treatment groups (####, *p* < 0.0001, ###, *p* < 0.001, and #, *p* < 0.05), and the gliben + exercise or gliben + IF differed significantly from the gliben-alone treatment group ($$, *p* < 0.01; $, *p* < 0.05). The gliben + exercise and gliben plus IF groups did not differ significantly.

The HFD group exhibited a significantly higher area percentage of positive TNF-α and iNOS immunological reactivity than the control and treatment groups (*p* < 0.0001; *p* < 0.01), as illustrated in Figure 13. In contrast to other treatment groups, the triple therapy group exhibited a significant decrease in the area percentage of positive TNF-α and iNOS immunological reactivity (*p* < 0.0001, *p* < 0.001, and *p* < 0.05). Additionally, the area percentage of positive TNF-α and iNOS immunological reactions was significantly lower in the groups treated with gliben plus either exercise or IF than in the group treated with gliben alone (*p* < 0.01; *p* < 0.05).

## 4 Discussion

Diabetic nephropathy (DN), a metabolic disorder, is a common side effect of both type 1 and type 2 diabetes. Even with the availability of effective drugs to treat diabetes mellitus, DN remains the leading cause of end-stage renal disease worldwide (Mestry et al., 2017). Numerous metabolic diseases, including diabetes, heart disease, and neurological problems, have demonstrated encouraging therapeutic results when treated with exercise and certain dietary practices such as IF. However, their effect in treating renal disease, particularly in DN, is still being studied. Meanwhile, our study emphasizes the remarkable efficacy of a triple therapy regimen consisting of gliben, IF, and exercise. This combined approach demonstrated significant improvements across various crucial parameters related to DN.

Type 2 diabetes is characterized by pancreatic beta-cell dysfunction and reduced insulin sensitivity. HFD is a known factor in developing insulin resistance. To accurately model type 2 diabetes in experimental settings, researchers often use moderate doses of STZ in conjunction with HFD. This approach creates a state of insulin resistance and partial beta-cell dysfunction, mimicking the human condition with significant increases in FBG and HOMA-IR index (Ohtsubo et al., 2011; Zhang et al., 2003; Srinivasan et al., 2005).

One of the most striking findings was the profound improvement in glucose metabolism. Triple therapy markedly reduced HOMA-IR and concurrently increased the insulin sensitivity index. This suggests a more efficient utilization of insulin by tissues and a significant reduction in tissue-level insulin resistance, leading to better glucose control. The individual contributions of gliben, IF, and exercise synergistically contribute to these positive metabolic outcomes, with the combined approach yielding the most pronounced benefits.

Untreated diabetic rats with DN often exhibit significantly altered lipid profiles, characterized by elevated levels of triglycerides and LDL cholesterol, coupled with lower HDL cholesterol. This condition, known as diabetic dyslipidemia, accelerates the progression of DN. The study found that all treated diabetic rats, especially those receiving triple therapy, showed marked improvements in their lipid profiles, indicating a crucial step toward preventing further renal damage. This aligns with previous research highlighting the importance of lipid management in DN (Suman et al., 2016).

According to the current study, HFD-induced diabetes often leads to a significant reduction in body weight due to hyperglycemia, hypoinsulinemia, increased muscle atrophy, and tissue protein loss (Cheng et al., 2013; Mestry et al., 2017). Triple therapy effectively prevented this hyperglycemia-induced damage, leading to a gradual increase in body weight in all treated groups. This suggests that the combined intervention helps preserve muscle mass and overall tissue integrity, which are vital for the general health of individuals with diabetes.

DN is characterized by a progressive decline in kidney function and structural alterations. Triple therapy in the current work demonstrated significant success in reversing these detrimental changes. A hallmark of deteriorating kidney function in diabetes is increased albumin and protein levels in the urine (albuminuria and proteinuria), coupled with a decrease in serum albumin concentration. Moreover, increased levels of BUN and blood creatinine, along with decreased excretion of creatinine in the urine, are well-established indicators of DN progression (Wang et al., 2011). This study observed that gliben administration, whether alone or in combination with IF and exercise, significantly lowered these levels, indicating a protective effect against DN. This reduction in urinary protein excretion is a crucial indicator of improved glomerular barrier function, reduced renal damage, and restoration of the kidney’s ability to filter waste products effectively, thereby preserving renal function.

Oxidative stress and inflammation are central to the pathogenesis of diabetic kidney damage. The triple therapy approach effectively targets these underlying mechanisms. Oxidative stress in DN is often characterized by a reduction in antioxidant enzymes such as GSH, CAT, and SOD, coupled with an increase in lipid peroxidation products such as MDA (Jeong et al., 2009; Ahmed, 2020). The study found that untreated STZ-induced DN rats exhibited decreased SOD, GSH, and CAT levels, while MDA levels increased. Importantly, triple therapy dramatically reduced MDA levels and significantly elevated antioxidant enzyme levels, indicating a robust antioxidant effect. The reduction in 8-OHdG, a marker of oxidative DNA damage (Sigenaga et al., 1989; Yuki et al., 2002; Wiseman and Halliwell, 1996), further supports the antioxidant potential of this combined therapy. Moreover, inflammation is a key contributor to chronic kidney injury and its progression in diabetes, often triggered by uncontrolled oxidative stress and the activation of stress-sensitive signaling pathways such as NF-κB (Wada and Makino, 2013; Ibrahim et al., 2020). Our study revealed that HFD/STZ-treated rats had significantly higher renal levels of pro-inflammatory cytokines such as TNF-α, IL-1β, and IL-6, along with reduced levels of the anti-inflammatory IL-10. These results are in line with a prior study by Guo L. et al. (2021). Triple therapy effectively attenuated this inflammatory response, likely by inhibiting the NF-κB pathway and consequently increasing IL-10 levels while reducing TNF-α, IL-1β, and IL-6. This anti-inflammatory action is crucial in preventing further renal tissue damage.

The polyol pathway and apoptosis are additional mechanisms contributing to DN. The polyol pathway, involving the enzymes AR and sorbitol dehydrogenase, plays a critical role in the development of DN (Zhou D. et al., 2020). Hyperglycemia increases the conversion of glucose to sorbitol, leading to its accumulation and subsequent cellular damage. This process also depletes NADPH, a cofactor for glutathione reductase, thereby exacerbating oxidative stress (Aghadavod et al., 2016; Dunlop, 2000; Sagoo and Gnudi, 2018; Yan, 2018; Huang et al., 2017). The current study found that gliben, alone or in combination with exercise and IF, significantly reduced the activity of AR and sorbitol dehydrogenase in the renal tissues of HFD-induced DN rats. This inhibition prevented sorbitol accumulation, thereby mitigating intracellular oxidative damage and supporting the idea that combination therapy can effectively reduce the polyol buildup characteristic of DN.

Hyperglycemia-induced oxidative stress and inflammation can lead to apoptosis (programmed cell death) in kidney cells, contributing to organ failure (Sapian et al., 2022; Singh et al., 2022). Diabetic rats showed elevated levels of the pro-apoptotic marker BAX and reduced expression of the anti-apoptotic marker BCL-2 in renal tissue; these results are in agreement with those of Guo L. et al. (2021) and Mohamed et al. (2021). Triple therapy significantly reduced BAX expression and remarkably increased BCL-2, indicating its anti-apoptotic potential. This preservation of renal cells is vital for maintaining kidney integrity and function.

Since TGF-β is known to be a regulator of renal fibrosis and to exacerbate kidney inflammation, there is substantial evidence that it plays a crucial role in the pathophysiology of DN. TGF-β, in the presence of hyperglycemia, can cause podocyte thickening, proteinuria, and diabetic nephropathy (Loeffler et al., 2011). Numerous previous studies have shown that TGF-β signaling is also significantly elevated in diabetic kidneys in both human and experimental animal models (Shaker et al., 2014; Wu et al., 2021; Wang, 2022). Consistent with previous studies, the untreated diabetic rats had considerably higher renal expression levels of TGF-β mRNA than the control group. However, gliben treatment protected HFD/STZ-induced DN animals from its harmful effects by restoring kidney TGF-β levels. The effects of gliben were stronger when combined with IF and exercise than when taken alone.

The role of inflammation extends to the production of nitric oxide (NO) and its impact on glomerular function. Increased production of pro-inflammatory cytokines such as TNF-α and IL-1β can lead to the activation of iNOS, particularly in renal cells (Narita et al., 1995). This overproduction of NO is implicated in diabetic hyperfiltration and glomerular abnormalities. Untreated diabetic rats showed increased iNOS expression in the renal cortex. However, triple therapy significantly decreased IL-1β and TNF-α levels, resulting in a marked reduction in iNOS expression. This suggests that the combined treatment can mitigate excessive NO production, thereby alleviating glomerular hyperfiltration and proteinuria.

Histological examinations revealed that diabetic rats exhibited segmental thickening of the glomerular basement membrane, mesangial matrix expansion, hypercellularity, distorted tubules, and inflammatory infiltration. Crucially, diabetic animals receiving triple therapy showed normal kidney architecture without tubular deformation. These histological improvements strongly support the biochemical findings and underscore the nephroprotective effects of the combined intervention.

In summary, the study highlights the potential of a combined therapeutic approach involving glibenclamide, intermittent fasting, and exercise in mitigating the progression of diabetic nephropathy, a leading cause of end-stage renal disease. Experimental models of type 2 diabetes, induced by high-fat diets and streptozotocin, revealed that triple therapy significantly improved glucose metabolism by reducing insulin resistance and enhancing insulin sensitivity. The intervention also alleviated diabetic dyslipidemia by lowering triglycerides and LDL cholesterol while elevating HDL cholesterol, thereby reducing risk factors associated with renal damage. Additionally, treatment groups demonstrated protection against hyperglycemia-induced muscle atrophy and weight loss, suggesting a broader systemic benefit in maintaining tissue integrity and metabolic balance.

Beyond metabolic regulation, the study found that triple therapy addressed key pathological mechanisms underlying DN. It reduced albuminuria and proteinuria, restored serum albumin levels, and lowered renal biomarkers such as blood urea nitrogen and creatinine, reflecting improved kidney function. Moreover, the intervention exhibited antioxidant, anti-inflammatory, and anti-apoptotic effects by enhancing protective enzymes (SOD, GSH, and CAT), suppressing pro-inflammatory cytokines, inhibiting polyol pathway activity, and shifting apoptotic markers toward cell survival. Importantly, triple therapy normalized TGF-β expression, reduced iNOS activity, and alleviated nitric oxide-mediated glomerular dysfunction. Histological analysis confirmed structural restoration of the kidney, with treated animals showing preserved glomerular and tubular architecture. Collectively, these findings underscore the synergistic nephroprotective potential of gliben, IF, and exercise in combating DN progression.

## 5 Conclusion

The triple therapy of gliben, intermittent fasting, and exercise shows strong synergistic effects by targeting multiple aspects of diabetic pathology. Gliben enhances insulin secretion, exercise improves sensitivity and glucose uptake, and fasting boosts metabolic flexibility while reducing inflammation. Together, these interventions provide comprehensive renoprotection by lowering apoptosis (reduced BAX and increased BCL-2) and suppressing TGF-β-driven fibrosis, thereby preserving renal integrity and preventing long-term damage in DN.

## 6 Future directions and clinical implications

The findings from this study strongly advocate for the potential clinical utility of combining pharmacological agents with lifestyle interventions in managing diabetic nephropathy. The synergistic effects observed suggest that an integrated approach could offer superior renoprotection compared to monotherapy. Future research should focus on translating these promising results into human trials to validate their efficacy and safety in diverse patient populations. Further investigation into the precise molecular mechanisms underlying the synergistic effects of gliben, intermittent fasting, and exercise would provide deeper insights into their combined power. Exploring optimal dosing regimens and intervention frequencies in human subjects will also be crucial for practical clinical application.

Furthermore, investigating the independent protective effects of IF and exercise on diabetic nephropathy through various pathways could be the focus of future research.

## Data Availability

The data sets analyzed during the current study are available from the corresponding author upon reasonable request.
